# Evaluation of a Complex Implementation to Shape, Spread, Scale, and Sustain a New Dementia Care Resource for Better, More Integrated Care

**DOI:** 10.5334/ijic.9872

**Published:** 2026-09-11

**Authors:** Celina Carter, Clementine Rotsaert, Paige Fernandes, Paul Holyoke

**Affiliations:** 1 SE Research Centre, CA

**Keywords:** dementia, caregivers, integrated care, quality of life, person-centred care, implementation

## Abstract

**Introduction:**

Researchers collaborated with experts-by-experience to create ‘Our Dementia Journey Journal’ (ODJJ), an interactive resource designed to enhance integrated care through communication and relationship building between caregivers and care providers. This study investigated how best to scale and spread the resource.

**Methods:**

ODJJ was implemented as a pilot in three communities with seven sites. A mixed-methods multi-phase sequential design with embedded formative and outcome evaluations was used, with experts-by-experience integrated throughout.

**Results:**

ODJJ was implemented serially in long-term care homes, First Nation communities, and South Asian communities. Implementation approaches and tools evolved through sharing experiences and tactics, with greatest uptake and use in the later sites.

**Discussion:**

The study confirmed that ODJJ can strengthen communication and relationship building, but how it was perceived, accepted and used for integrated care differed. The cumulative results generated a spread and scale (marketing) strategy, with messaging, materials and supports to facilitate uptake and use across Canada.

**Conclusion:**

Pilot implementation for integrated care resources with a strong, complex evaluation can lead to a detailed plan for spread and scale.

## Introduction

Person- and family-centred care is a widely recognized approach for ensuring quality dementia care [1]. Persons living with dementia (PLWD), family/friend caregivers and healthcare providers describe this approach as “promoting a continuation of self and normality” [2]. Caregivers play an integral role including acting as essential sources of intimate knowledge about PLWD [3,4].

However, there are challenges with integrating caregivers in the dementia circle of care; they are often excluded from decision-making and changes in care [5]. Increased communication and better relationships between caregivers and care providers [6,7,8] can result in increased clarity of their respective roles. Role clarity, including changes over time [9,10], can contribute to lower levels of burden on caregivers and improved mental health, wellbeing, and quality of life [11]. Similarly, with more information exchange and better relationships, the quality of life, mental health and wellbeing of PLWD can increase [12,13].

Researchers in the SE Research Centre (SERC) collaborated with the Alzheimer Society of Canada and experts-by-experience [14], and they confirmed that caregivers and care providers are not well supported to exchange knowledge and skills, to sustain relationships, and to achieve health-related outcomes. A resource intended to fill these gaps, developed in full collaboration with experts-by-experience, is called Our Dementia Journey Journal (ODJJ). The development process is described elsewhere [14].

The ODJJ was then adapted with culturally relevant imagery and wording for First Nation and South Asian communities (specifically for Punjabi- and Hindi-speaking users) since experts-by-experience indicated that there were few relevant dementia resources in those communities and that there is a stigma about dementia that prevents it being talked about, acknowledged and addressed.

**Figure 1:**
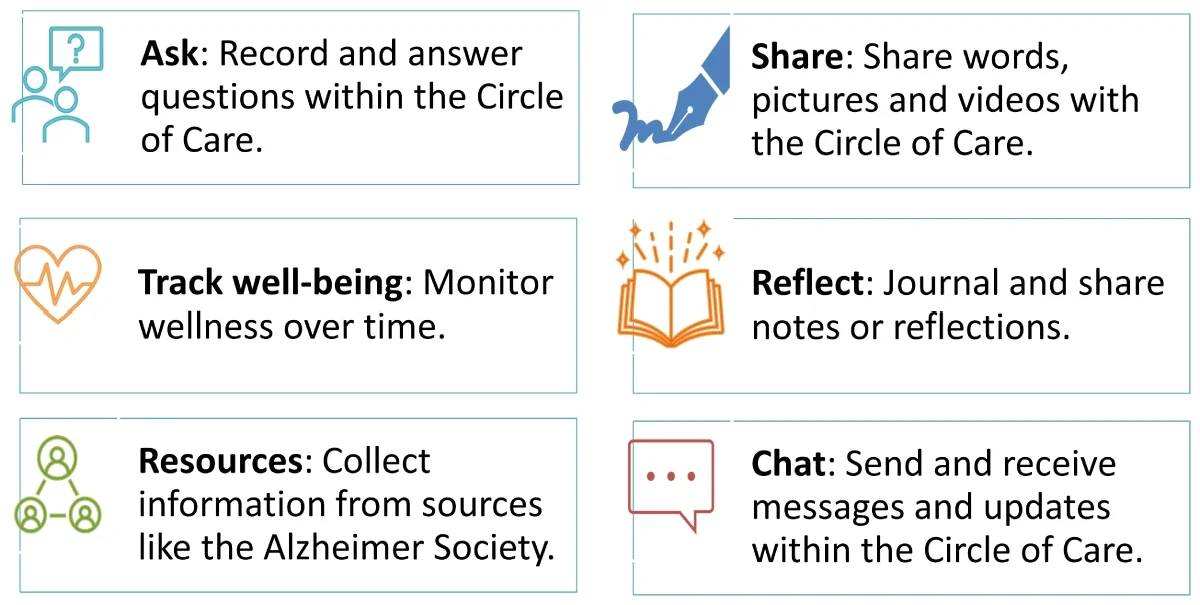
The six features of the ODJJ resource.

The ODJJ is an interactive resource with paper-based and mobile app versions [15], and is comprised of six features that can be used flexibly to meet the needs of the users (Figure 1).

The overall goal for the work reported here was to identify how best to spread and scale the ODJJ, and to generate a spread and scale (marketing) plan for diverse health and social care settings. The questions posed were:

1. What implementation approach and activities are effective to create interest in, and encourage people to use, the ODJJ in different community types?
  - What components of the implementation approach were successful, and which required adjustments?
  - How do people use the ODJJ resource?
2. What do ODJJ users experience in terms of increased knowledge and skills, caregiver and care provider relationships, and health-related outcomes?

## Evaluation Methods

### Project governance

Experts-by-experience were primarily engaged through a pan-Canadian Project Advisory Committee (PAC). The PAC was established in January 2024 and accrued membership as the ODJJ was introduced into communities. The PAC met monthly online to oversee and inform project progress and exchange implementation experiences and tactics. By April 2025, the PAC consisted of over 20 people including PLWD, caregivers, healthcare providers, and leaders from several communities.

### Settings

The community settings were aligned with communities involved in the ODJJ development and adaptations: long-term care homes (LTC), First Nation communities, and South Asian communities. The ODJJ was delivered in seven sites across three communities (Table 1).

**Table 1:** Description of delivery sites.


LTC COMMUNITIES	FIRST NATION COMMUNITIES	SOUTH ASIAN COMMUNITIES

Site 1: Calgary, Alberta. 20-bed dementia care unit. Site 2: Red Deer, Alberta. 24-bed dementia care unit.	Sites 1, 2 and 3: Robinson-Huron Treaty area on the North Shore of Lake Huron in Ontario, represented by the Mamaweswen North Shore Tribal Council. The sites have 140–1,000 Band Member residents.	Site 1: Brampton and Mississauga, Ontario. Adult Day Program. Site 2: Brampton, Ontario. Organization offering support to PLWD and their caregivers.


### Implementation approach

**Figure 2:**
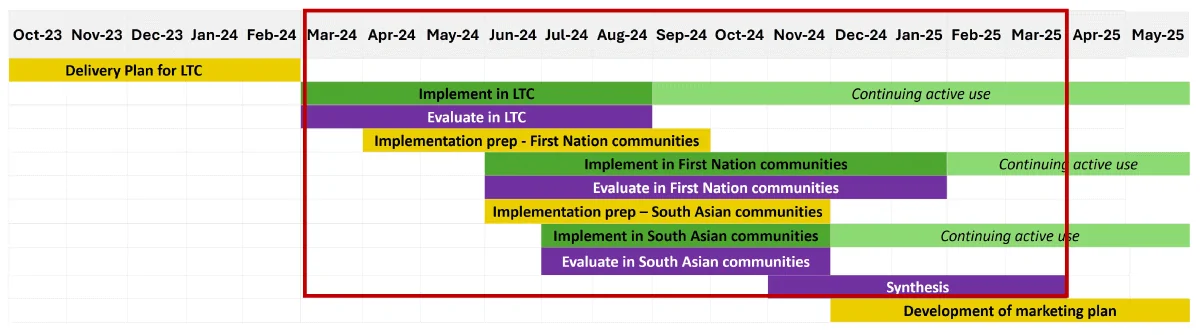
Project timeline.

The ODJJ was implemented between March 2024 and January 2025 sequentially as a pilot in each community (Figure 2). The pilot period was four months, believed during planning to be appropriate to observe the introduction of the ODJJ to the community (1 month), ongoing user uptake (1 month), and substantial use or non-use (2 months). (As it happened, in the First Nation communities, the timeline was extended to eight months due to absences of key personnel). A three-person SERC Delivery Support Team coordinated the implementation, and collaborated with Site Delivery Teams composed of leaders and/or community champions from each site.

The first implementation, in LTC, was supported by a service design company that developed a Delivery Plan. Based on a short LTC “residency,” the service designers made six key recommendations for ODJJ implementation in LTC: a) use clear messaging: why and what; b) provide implementation tools and support; c) ensure leadership buy-in; d) identify and recruit early adopters; e) support the use of the ODJJ; and f) develop feedback mechanisms.

The SERC Delivery Support Team developed initial tools, resources and ideas based on this Plan. The tools and resources were available in English, Hindi, Punjabi and First Nation versions. The SERC Delivery Support Team offered each community and individual users the choice of implementing and using the mobile app or a paper version in a binder. With guidance from the Site Delivery Teams and the PAC, the implementation approach evolved for First Nation communities as experience was gained in the LTC sites and evolved again for South Asian communities as experience was gained in First Nation communities. Implementation adaptations included refining tools and resources, offering customized introduction sessions, creating modifiable poster templates to invite users, offering regular check-ins, developing an e-learning module for care providers, developing an implementation process guide for community leaders, offering coaching sessions for downloading and setting up the app, and gathering feedback to inform app upgrades to enhance usability and performance.

### Evaluation design

The SERC Evaluation Team (two Research Scientists and a Research Assistant) designed a mixed-methods multi-phase sequential evaluation [16].

To answer question 1, a formative evaluation of the implementation approach in each community was conducted. To answer question 2, an outcome evaluation was conducted to determine the effect of the use of the ODJJ. The evaluations were conducted in three stages aligned with the communities, and in a fourth synthesis stage (Figure 2).

The formative evaluation focused on ten Consolidated Framework for Implementation Research (CFIR) constructs [17] to structure data collection and analysis about implementation successes and adaptations (Table 2).

**Table 2:** Alignment of Formative Evaluation Questions with CFIR Constructs and Data Sources.


FORMATIVE EVALUATION QUESTION	CFIR CONSTRUCTS [17]	DATA SOURCE

What components of the implementation approach were successful, and which required adjustments?	Individual Domain Opportunity to implement ODJJ Capability to implement Motivation to implement Implementation Process Domain Facilitators & barriers to implementation (Assessing context) Roles & goals (Planning) Tailoring strategies Attracting & encouraging participation (Innovation recipients) Inner Setting Domain Tension for change Mission alignment Compatibility	Delivery Team debriefs Pre-post Surveys Interviews Focus Groups

How do people use the ODJJ resource?	—	Pre-post Surveys Interviews Focus Groups


The outcome evaluation focused on variables of interest from the literature and confirmed by experts-by-experience during the development of the ODJJ [14]: increased caregivers’ dementia-related knowledge and skills [3,4,18]; improved relationships between care providers and caregivers [6,7,8]; improved quality of life for caregivers and PLWD and improved wellbeing or other health outcomes [12,13].

### Data collection

#### Delivery Team debriefs

The SERC Delivery Support Team hosted regular check-in meetings at an agreed upon time (weekly, bi-weekly, monthly) with the Site Delivery Teams, recording minutes to capture implementation approaches and activities to create interest in the ODJJ and to encourage use. The SERC Evaluation Team reviewed the minutes and conducted pre/post pilot debriefs with the SERC Delivery Support Team to answer question 1.

#### Pre- and post-surveys

ODJJ users were invited to complete surveys related to both questions 1 and 2. Pre-surveys collected outcome data on communication, quality of life for caregivers and PLWD, and demographics. Post-surveys collected the same data as well as data on ODJJ helpfulness and skills and knowledge to address question 2, and usage and overall experience to address question 1. Quality of life was measured using the 13-question Quality of Life–Alzheimer’s Disease (QOL-AD) measure for PLWD [19,20], and the 30-question C-DEMQOL for caregivers [21], each (including the C-DEMQOL subscales) shown to have strong psychometric properties [19,20,21].

#### Interviews and focus groups

Semi-structured interview and focus group guides were created to collect data to answer question 1: use and non-use, frequency of use, factors supporting use, and how to make the ODJJ easier to access and use. The interviews and focus groups also addressed the outcome question about relationships and wellbeing. ODJJ users were invited to participate in interviews or staff focus groups at the end of each pilot period. Interviews and focus groups lasting 50 minutes on average were conducted in person or using MS Teams and were recorded and transcribed verbatim.

### Data analysis

The SERC Evaluation Team employed multiple analytic techniques. Qualitative data was analyzed using a deductive thematic framework [22] drawing on the CFIR constructs (Table 2). After initial coding, the Evaluation Team came to consensus about the categorization of data and generated a descriptive summary of each category describing the facts of what happened in sequence and what this meant for participants [23]. Preliminary findings were discussed at monthly PAC meetings to shape evolving implementation approaches across sites. The PAC was instrumental in identifying ways to modify the implementation approach for optimal integration into workflows across sites [17]. Quantitative data from the surveys was analyzed using descriptive statistics, Shapiro Wilk tests for normality, paired t-tests, and Cohen’s d for effect size. As recommended by Banerjee & Daley [24], mean substitution was used for missing data if five out of six C-DEMQOL items were answered in each subscale. When all three settings’ data had been analyzed, a synthesis was done by the Evaluation Team by comparing them [25].

### Ethics

The Southlake Regional Health Centre’s Research Ethics Board waived the requirement for an ethics review of the study protocol (S-038-2324) because it was a program evaluation, falling outside scope of “research” [26]. Nonetheless, the Tri-Council Policy Statement: Ethical Conduct for Research Involving Humans – TCPS 2 (2022) [26] requirements were followed.

## Results

### Context and demographics

The Site Delivery Teams were comprised of members in Table 3. All were women except one man in LTC Sites 1 and 2.

**Table 3:** Site Delivery Team members.


SITES	SITE DELIVERY TEAM MEMBERS

LTC Site 1	Site Director, Resident Care Manager, Clinical Educator, Director of Care, Therapeutic Recreation Manager

LTC Site 2	Site Director, Clinical Educator, Wellness Manager, General Manager

First Nations Sites 1, 2, 3	FNIM Program Community Engagement Liaison, Community Champions (Elders), Director of Home and Community Care

South Asian Site 1	Two Health Service Managers, two Client Care Counselors, Psychogeriatric Resource Consultant

South Asian Site 2	Psychogeriatric Resource Consultant (from Site 1), Senior Manager of Health Promotion and Prevention


Most caregiver T1 survey responses (n = 25) came from South Asian sites (72%), followed by LTC (16%) and First Nation communities (12%). Caregivers were mostly women (72%), children (62%) or spouses of PLWD (33%), and more than a quarter were over 70 years of age (36%). Six PLWD participated (equal gender distribution, 83% over 70, most living at home). Twenty-four care providers participated in the T1 survey, mostly women (79%) providing community care.

Focus groups included 15 providers from across LTC Sites 1 and 2, and South Asian Site 1. One provider from South Asian Site 2 was unable to attend the focus group, so participated in an interview. Most identified as women (86%), about half worked in LTC and half in community-based organizations, and 10 were users of the ODJJ.

Interviews were conducted with 14 caregivers across all sites, including 11 ODJJ users. Most identified as women (71%). There was diversity across ethnicity including South Asian (42%), white (36%) and First Nation (14%).

### Formative evaluation results

The Evaluation Team examined the formative evaluation data at baseline, throughout each pilot, and at each pilot end. First Nation sites’ data is amalgamated to prevent identification.

#### ODJJ implementation motivation, opportunity, and capability

According to team debrief data, motivation to implement the ODJJ was high across all Site Delivery Teams for various reasons including meeting expressed needs of Family Council members, alignment with the Eden Alternative philosophy of care [27], improving care for First Nation community members, building a supportive dementia community to address stigma, and connecting people to additional dementia resources.

However, despite high motivation at baseline, LTC Site Delivery Teams had capability but low opportunity to use the ODJJ due to limited autonomy in the highly regulated setting, and limited staff availability. First Nation Site Delivery Teams (Community Champions) had high opportunity with autonomy in their role, and capability with knowledge of caregiver needs. South Asian Site Delivery Teams had medium opportunity due to limited time and competing priorities, with capability in this flexibly structured environment.

As the pilots progressed, opportunity (e.g., availability) for implementation for some Site Delivery Teams was negatively impacted by staff turnover and reassignment. At South Asian Site 1, one of the two Adult Day Program managers left, reducing capacity to support the delivery. Limited availability appeared to decrease confidence in teams’ ability to deliver. When staff had a good understanding of how the ODJJ could support potential users’ needs, motivation increased, with the inverse also being true. However, over the pilot periods, there was continued engagement in the PAC by Site Delivery Team members keen to share learnings and promote the ODJJ.

Within Site Delivery Teams, specific individuals acted as naturally occurring champions with strong motivation. Natural champions were crucial for success but designating a person as a champion was different from champions putting themselves forward as such. In one site, the natural champion took a leave of absence, and their replacement was not as committed to the ODJJ, which was then deprioritized. Similarly, if there is motivation but not opportunity, champions were less successful.

#### ODJJ site tension for change, mission alignment, and compatibility

At baseline, tension for change (i.e., the degree of challenges the ODJJ could address) varied across sites according to team debrief data. The Site Delivery Team from the First Nation sites and South Asian Site 2 explained they had a high tension for change due to limited access to dementia care supports, few culturally competent dementia resources, caregiver isolation, and stigma preventing discussions about dementia. South Asian Site 2 Delivery Team said they had medium tension for change as their program was already working well; however, there was a need for culturally relevant dementia care resources, more caregiver-care provider connections, and even better care for PLWD.

According to the LTC Site Delivery Team, caregivers, leaders, and staff felt different levels of tension for change. There was high tension for change among caregivers due to burnout, lack of connection with staff, and a desire for person-centred care; medium tension for change among leadership who wanted staff to be more person-centred; and low tension for change for most care providers as they felt they were delivering good dementia care. As the pilots progressed, tension for change in LTC increased. Providers in LTC gradually saw that the ODJJ could increase their knowledge about PLWD and help them to better support residents’ expressive behaviours, a challenging part of their work.

The ODJJ aligned with missions (goals) of the sites, such as supporting a person-centred approach to dementia care and the Eden Alternative philosophy of care [27], increasing caregiver support, offering meaningful resources to caregivers, spreading awareness of dementia, and improving communication. There was compatibility of the ODJJ with processes in First Nation sites (e.g., a tool in caregivers’ homes) and South Asian Site 1 (e.g., a communication tool within the Adult Day Program). However, there was uncertain compatibility in LTC and South Asian Site 2. For example, LTC Site Delivery Teams were unsure how ODJJ would fit with providers’ workflow, how to distinguish the ODJJ from clinical charting, and how to address privacy concerns.

The more regulated circumstances of LTC made integration of the ODJJ into existing clinical workflows a challenge. Privacy concerns also prevented ease of access to the ODJJ in this setting. Eventually, introducing the ODJJ to new rather than existing residents and caregivers was identified as a quality improvement initiative to highlight in preparation for facility accreditation. In contrast, it was first believed that introducing the ODJJ to caregivers in the flexible home care environment in First Nation communities would be successful; however, without any structure to encourage the circle of care to use the ODJJ, sustained uptake was low. Similarly, caregivers in South Asian Site 2 lacked a structured circle of care, resulting in lower level of uptake.

#### ODJJ implementation facilitators & barriers, roles & goals, tailoring strategies, attracting and encouraging participation

At baseline, there were many anticipated facilitators and barriers to implementation, few clear roles and goals for implementation, some tailoring strategies and various plans for attracting and encouraging participation (Table 4).

**Table 4:** Baseline implementation plan for each site.


SITES	FACILITATORS & BARRIERS	ROLES & GOALS	TAILORING STRATEGIES	ATTRACTING & ENCOURAGING PARTICIPATION

LTC Sites 1 and 2	Primary facilitators: LTC Site 1: a collaborative team. LTC Site 2: Commitment to person-centred care Anticipated barriers: staff workload, caregiver burnout, privacy concerns, and technology security.	No clear roles and responsibilities and no implementation milestones or goals were documented.	ODJJ orientation e-learning module available for staff. ODJJ was in a binder in the rooms of PLWD.	In-person ODJJ orientations with 20-minute presentation for caregivers and staff. Promotional materials included posters, newsletters, and a display board.

First Nations Sites 1, 2, 3	Primary facilitator: Trust in the Site Delivery Team. Anticipated barriers: fear of talking about dementia, leadership challenges, decreased community visiting, and historical factors.	The roles included as-needed support from Community Champions, and the FNIM Liaison checking in throughout the pilot. The goal was to support even just one person.	1:1 personalized, ODJJ orientations with relational conversation to build trust with caregivers. Caregivers provided with First Nation version in binders in their home.	Community Champions recruited potential users. ODJJ orientation was a 2-hour culturally safe “tea and talk” focused on caregiver needs. Promoting use was primarily by word-of-mouth.

South Asian Site 1	Primary facilitator: Existing daily connections. Anticipated barriers: caregiver burnout, staff turnover, and dementia stigma.	Lack of clear roles and responsibilities for connecting with caregivers to support use. No milestones or goals recorded.	All resources and materials were provided in English, Hindi, and Punjabi. The ODJJ was used to communicate about activities in Adult Day Program.	A 2-hour in-person ODJJ orientation session for caregivers first. Staff were oriented through online orientation videos. Promotional materials included posters, display board, and reminders at meetings.

South Asian Site 2	Primary facilitator: Commitment to being a supportive dementia community. Anticipated barriers: caregiver burnout, staff turnover, dementia stigma, and limited human resources.	No clear roles and responsibilities and no implementation milestones or goals were documented.	All resources and communication materials were provided in English, Hindi, and Punjabi. Volunteers’ involvement supported use and spread.	A 2-hour in-person ODJJ orientation session for caregivers first. Staff were oriented through online orientation videos. Promotional materials included posters and phone call reminders.


As pilots progressed, Site Delivery Teams said implementation challenges included staff turnover, competing priorities, unclear roles and responsibilities, and lack of clear milestones. Despite some facilitators, at both LTC sites there were changes to funding, new regulations and accreditation requirements, and communicable illness outbreaks that limited implementation success. Similarly, in the First Nation communities, caregivers becoming sick, external stressors like family bereavement, and increasing caregiving workloads limited implementation success. To address these barriers, Site Delivery Teams recognized the need for clearer roles and responsibilities in rolling out and sustaining use of the ODJJ and for clearer milestones and end goals. Some delivery sites, such as South Asian Site 1 and LTC Site 2 did this by the end of the pilot periods.

Tailoring strategies to address barriers happened over the course of some implementations. South Asian Site 1 was successful in promoting the ODJJ as a way for staff and caregivers to share photos and messages regarding the Adult Day Program. Likewise, by the end of the pilot, LTC Site 2 had incorporated the ODJJ into their resident admission process.

Attracting and encouraging participation was a challenge. While there was buy-in and agreement that the ODJJ could be helpful, this alone did not lead to widespread uptake. Several Site Delivery Teams realized a need for universal and rolling orientations with staff, champions, and providers to ensure knowledge and confidence in ODJJ use. In LTC, the promotion of the ODJJ did not reach many caregivers and there was confusion about how to become involved with the ODJJ. In the First Nation communities, caregivers did not request support to use the ODJJ but had limited follow-up and structure, resulting in low uptake. At the South Asian sites, additional strategies were developed to offer caregivers support including mass emails to spread awareness and establishing a ‘help desk’.

#### ODJJ use across sites

Reflecting the gradual learning about the best approaches for implementation, the number of completed surveys and interest in participating in interviews and focus groups increased over time. The highest participation group was users in the South Asian sites. While only two LTC caregivers completed the pilot phase and pre/post surveys, 18 South Asian caregivers completed both.

Data showed that all ODJJ features were of interest and used but rarely did individual users use all features. As noted above, particularly in LTC and the First Nation sites, incomplete reach of the ODJJ to the entire circle of care tended to limit regular and extended use. However, the potential helpfulness of each feature was emphasized in the (limited) survey results with a majority (62% to 90%) of caregivers and a majority of providers (60% to 90%) indicating each feature was helpful, which was confirmed in interviews and focus groups.

ODJJ users said the resource was helpful for efficiently sharing information among the circle of care members. Caregivers in the LTC sites discussed how the “ask” function (see Figure 1) could be helpful for receiving updates from staff. One Site 1 caregiver shared, “… having an app on my phone and to be able to fire a question off to the staff … as opposed to calling … just day-to-day [to see] how she’s doing”.

The same caregiver felt the share feature that allows videos could also be helpful, stating:

“[the continuing care home] brings in singers every now and then … I just thought of this, through the app, is there ways for the [staff] to take a 30 second video or something and communicate … pictures are always great.”

South Asian Site 1 caregivers and providers discussed the helpfulness of the communication features in the ODJJ for documenting, tracking, and communicating care needs between caregivers and staff. A caregiver shared,

“I could share [in the chat function of the app], ‘His blood sugar was really low this morning and he didn’t eat much breakfast and he’s kind of in a mood and he’s a bit grumpy’. And I could put that in and they would know that and they would deal with it”.

Another South Asian Site 1 caregiver explained why “reflect” and “chat” features were helpful:

“…the great thing with the app … sometimes information is said and it’s not remembered or, depending on who I tell it to, the cool thing about the app, it’s documented and they can go back and refer to it.”

One LTC Site 1 caregiver agreed stating:

“I think there’s really high potential families would be doing stuff out of the home and then the caregiving team inside the home is doing stuff and there’s for sure that risk [of] a disconnect”.

The other most commonly discussed feature was “share” which included the tools ‘All about me’ and ‘Top 5 things’. Caregivers in the LTC sites said these tools could help with communicating the PLWD’s personal interests and preferences to sustain their personhood. One LTC Site 2 caregiver stated, “My mom was still playing the piano, and it was things like that. When there’s musical entertainment in there, I don’t ever want her to miss it.”

LTC providers agreed, with one provider from Site 1, saying, “… do it [ODJJ] on admission then we have a little bit something to know about that resident when they come in.” The LTC site providers were especially interested in the potential for this feature to help them respond to residents’ expressive behaviours, sharing:

“I mean, we have such drastic behaviours. … I think it would just be beneficial to help and assist … how we can handle them better.”

First Nations caregivers also thought the “share” feature could be helpful to orient care providers to the preferences and abilities of PLWD. One caregiver shared:

“If we get new PSWs there’s nothing to look at, to see, we just got to sit there and go over and over what she likes and what she dislikes but this is a good tool for it.”

### Outcome evaluation: knowledge and skills, relationships, and health-related outcomes

#### Improved dementia-related knowledge and skills

Most ODJJ users talked about the benefit of improved dementia-related knowledge. A LTC Site 1 caregiver talked about their increased knowledge in recognizing the personhood of the PLWD and developing skills in person-centred care after using the ODJJ, stating:

“I get the sense sometimes it’s easy to think that our family members and patient doesn’t know what’s going on anymore. And I think that’s true some of the time, but I feel like the sentiment is still there, […] they may not know what’s going on, but every once in a while, it’s nice to look down and know your nails are done or your hair is brushed”.

The FNIM Liaison reiterated that she heard caregivers talking about this same benefit. She explained:

“… one woman … who was there with her mom who had dementia. And she said, ‘my mother is very triggered by dishes in the sink’. She doesn’t like dishes in the sink. … if I could just make sure the PSWs know that’ … they would use the ODJJ … to make sure that anybody coming in the house, as a priority and part of their orientation, would review that ODJJ … to learn about who they’re caring for.”

South Asian site users also described how the ODJJ helped to improve knowledge and skills for providing person-centred dementia care by shifting from being paternalistic to focusing on what the PLWD values and wants. A Site 1 & 2 care provider explained:

“Sometimes people when they are [new] into caregiver role, they don’t know what to expect. … they think ‘I know the best’ and the person who is receiving the care, that person doesn’t know anything … Sometimes being caregivers, we tend to forget what our loved ones want. … it’ll give an opportunity for the caregivers to reflect on the needs of their loved ones. So, what they want, what does mom want, what does dad want? What are the questions I should be asking?”

The ODJJ sparked conversations that helped reduce stigmatization among this cohort of the South Asian community. A Site 1 care provider said:

“Definitely, [the ODJJ is] increasing awareness about this disease because it’s a new disease for South Asian community, and there’s a lot of stigma attached to mental health and neurocognitive disorders.”

#### Strengthening relationships within and beyond the circle of care

Caregivers said the ODJJ helped them trust staff by feeling the needs of the PLWD were being met as facilitated by increased information sharing. One LTC Site 2 caregiver stated:

“… when these guys know about her needs, and what I like and don’t like, then it’s better for me to know that everybody’s on top of it.”

Similarly, for South Asian users, having transparent conversations through the ODJJ helped foster trust. A Site 1 provider stated:

“I think the caregivers are happy that this communication is going and that they’re seeing what the clients are doing here in the program”.

The ODJJ also strengthened community support beyond the circle of care through connecting people and starting conversations. The FNIM Liaison shared how important the ODJJ can be for opening conversations that lead to more support. She explained how this happened:

“…when [caregiver] started talking about how lonely she was, how scared she was, [Community Champion] was sitting there and looking and saying, ‘I had no idea. Like I just had no idea that that you were feeling this way. And now that I know that I can really help, I can rally support. We can do things to support you and make sure that you’re cared for and that you and [PLWD] have visitors.”

The ODJJ also strengthened South Asian caregiver-to-caregiver support. The ODJJ orientation and training brought caregivers together, building an ODJJ support group. This group used the ODJJ as a conversation starter about the challenges they were experiencing as caregivers. One South Asian Site 1 caregiver explained:

“Everyone feels like you’re alone in this whole thing, right? … when we start talking about resources that are available, then everybody sits up and listens. And, it’s good to know how each one manages their day… this app also kind of draws us closer together.”

This support group acted as “a safe forum where [caregivers] network with each other” and get ideas about “better solutions” to their “similar challenges.”

#### Quality of life and wellbeing

Many caregivers explained that the ODJJ supported their quality-of-life by inviting self-reflection. South Asian caregivers talked about reflecting on the caregiving journey, which helped wellbeing. One caregiver from Site 1 said:

“I have an outlet now where I can voice my thoughts instead of just sitting at home and saying, ‘Shit. What the hell? How do I handle this?’ At least now I have the outlet where I can journal-entry and it helps.”

Likewise, a First Nations caregiver talked about how the ODJJ invited reflection on needs for self-care and support, sharing:

“… so not only does it focus on the caregiver and what works for you but also I noticed that it also for the caregiver as myself and then how it reminds me that I need to take care of myself.”

LTC care providers echoed how they thought the ODJJ would support caregiver wellbeing by having a place to share worries. One provider from Site 2 stated:

“… we have to make a difference in these people’s lives and help walk them through this journey, … I see a really great opportunity here with this [ODJJ] … a lot of families come into this, and they really don’t understand what’s happening and what’s going on … being able to write down what their worries and their concerns … allows them that opportunity then to put it down and know that we’re able to go back and review that”.

We were unable to determine if the ODJJ influenced the quality of life for PLWD as we only received three completed time 1 and 2 QOL-AD surveys. To understand whether the ODJJ influenced caregiver quality of life, a series of paired t-tests were run examining the C-DEMQOL total score and subscales between timepoints (see Table 5). The data met the assumption of normality, and there were no significant outliers present. Given the exploratory nature of the work, uncorrected p-values are reported.

**Table 5:** Descriptive Statistics for the C-DEMQOL scale.


MEASURE	TIME 1		TIME 2		DIFFERENCE	COHEN’S *d*
				
*N* = 18	MEAN	SD	MEAN	SD	MEAN	*dz*

C-DEMQOL Total*	66.00	6.90	83.60	19.50	17.59	0.82

Personal Needs*	2.32	0.45	2.80	0.85	0.48	0.50

Wellbeing	2.27	0.43	2.58	0.87	0.31	0.35

Relationship with PLWD*	2.15	0.37	3.20	0.10	1.05	0.86

Confidence in Future	2.26	0.41	2.48	0.71	0.22	0.33

Feeling Supported*	2.07	0.45	3.04	0.94	0.97	0.90


Data showed that the caregivers who used the ODJJ demonstrated a statistically significant increase in overall QoL *t*(17) = 3.4, *p* = .003. Examination of the subscales demonstrated a significant increase on the subscales measuring the relationship with PLWD, *t*(17) = 3.65, *p* = .002, feeling supported, *t*(17) = 3.69, *p* = .002, and meeting personal needs, *t*(17) = 2.12, *p* = .049. There were no statistically significant improvements on the subscales measuring changes to wellbeing *t*(17) = 1.49, *p* = .154, or confidence in the future *t*(17) = 1.37, *p* = .189.

### Synthesis of findings

Prompting initial interest in the ODJJ was not difficult in any setting or at any level – leaders, managers, and care providers and caregivers. Across the very different settings in this project, the ODJJ aligned well with organizations’ missions. Further, leaders in the organizations were motivated to implement the ODJJ to respond to calls for integrated dementia care that strengthens relationships and promotes person-centred dementia care [28].

These motivations generally mirrored a tension for change. This is consistent with findings in other studies where mission alignment and achievement of organizational goals contribute to implementation success [29,30]. But as the evaluation results show, the tension for change can vary over time, as different priorities arise, including disease outbreaks, funding changes, and regulatory demands.

Similarly, the opportunity to implement the ODJJ varied across settings and over time as external influences exerted pressure. While some implementations appear to have failed early, the findings highlight what others have found in implementation studies [31]: initial experience may not determine long term experience. Here, cumulative learnings resulted in the most participation in the later delivery sites. Initial implementation disappointments in LTC did not seem to predict future success; both LTC sites turned implementation challenges into learnings about tailoring strategies, roles, motivators, and ways to address compatibility issues.

Compatibility of the ODJJ was seen as an issue in almost all situations, as existing workflows and processes appear to have prevented the very changes that leaders and others were motivated and mission-driven to make. Nonetheless, when even one ODJJ feature was experienced as beneficial, users discovered how to incorporate it into practice. This reflects the experience in other studies when the ‘why’ of an innovation aligned with professional values [32] or dissatisfaction with current processes [33] and in different ways for different people [34].

Capabilities to implement varied across settings, and sometimes, when there was high capability, external pressures had less effect. Also, as others have shown [34], when naturally occurring champions were involved, capabilities rose as they identified paths to attract and encourage successful uptake and use.

Interestingly, although using the ODJJ was perceived to have, and was shown from limited data to have, positive effects on health outcomes, all users including managers and leaders, were more interested in the instrumental uses of the ODJJ, its function in the day-to-day. This reflects, perhaps, the greater influence of mission alignment and motivation to improve process.

## Discussion

After an extensive development process with deep and authentic engagement with experts-by-experience, [14] the ODJJ was delivered and measured in three communities in seven sites across Canada, each working in different circumstances, regulatory and governance structures, and funding arrangements. The use and benefits of the ODJJ in real world settings aligns with calls for integrated dementia care for PLWD, and for more knowledge and skills, improved relationships and better health outcomes for PLWD and their caregivers [28]. There was high motivation to deliver the ODJJ across sites, with a range of motivators related to the missions of each community.

While tension for change varied over time, significant issues of implementation capabilities and of compatibility with workflows and processes were experienced in all settings [5,35]. Perhaps most strategically important for future spread and scale, the results point away from a one-implementation-fits-all approach and towards emphasizing multiple ways to implement and use this resource to support the integration of caregivers into dementia care [5,35]. Further, initial implementation failure does not necessarily portend failure later, as others have discovered [31]. A variety of tools [5,35], resources and supports were developed and refined over time by the SERC Delivery Support Team to address the real-world implementation challenges faced by Site Delivery Teams all of whom had varying implementation capabilities and success.

As a result of the findings, a structured, multi-tiered marketing strategy incorporating multiple tools, resources and supports developed by the SERC Delivery Support Team for specific organizational contexts and user needs was developed and tested by sites and reviewed by the PAC for relevancy [36]. The marketing materials emphasize the value proposition of the ODJJ as an evidence-based communication and relationship building tool between caregivers and care providers, as well as its potential to reduce the stigma of dementia, and though originally unexpected, to encourage more caregiver support from other caregivers or community members. Importantly, the marketing materials clarify that while the ODJJ enhances person-centered care and improves caregivers’ quality of life, it supplements rather than replaces hands-on care delivery—an important distinction for managing expectations during broader implementation efforts.

Gaps in capabilities to implement necessitated different ways of encouraging uptake; accordingly, materials and tools in the marketing strategy provide tactical advice and tools (e.g., sample posters, social media campaign materials, orientation tools). Furthermore, perceptions of incompatibility of the resource within diverse settings at the site or user level have been addressed by signaling that the ODJJ’s different functions can be used separately or together, depending on workflows and patterns of care. Different guides for implementation for organizations with health and social care providers and for caregiver groups were developed that encourage role clarity and goal setting [17], and active use of newly available implementation opportunities.

Observations of the different expressions of interest in adoption and use of the ODJJ have supported the development of different, alternative marketing messages for potential implementers at all levels (e.g., organizations, leaders, managers, care providers). The marketing strategy reflects the differences also by differentiating between organizations that will amplify awareness through website posting or community outreach; implementation-focused organizations and caregiver groups that require comprehensive onboarding support including implementation guides, regular check-in meetings, and ongoing evaluation protocols; and, direct end-users who will be supported primarily through web-based resources and email support systems rather than direct engagement.

This systematic marketing approach that enables scale and spread of the ODJJ for integrated care will be supported by the continued commitment by the PAC members who will share experiences and advice to support new adopters to address implementation challenges in diverse sites and community contexts.

## Limitations

Because uptake and use rates increased as new sites were introduced to the ODJJ, rich data accumulated about what worked and what did not. However well intended including such varied settings as LTC, First Nation communities and South Asian communities was, all the data came from organizations and people who saw value in the ODJJ. This raises a concern about generalizability, however the variety of deeply-engaged people and perspectives involved in the ODJJ’s development [14] as well, and their contribution to making the ODJJ highly aligned with goals of person- and family-centred care, one of the strongest facilitating factor across all sites, makes the results transferable. Further, with respect to the quantitative findings, the participants who completed the surveys may have been limited to those who benefited from the ODJJ, and therefore motivated to express their views in the surveys. This study is also subject to the limitation that the care provider and caregiver samples were predominantly female, which may limit the generalizability of the findings to male care providers and caregivers. Further, the limited data on ODJJ users prevented a detailed analysis of how intersectionality impacted ODJJ use and outcomes.

Future research opportunities arising from this study include confirming that the multi-level ODJJ marketing approach results in better integration of caregivers and care providers of PLWD, including where there is a greater proportion of men involved than in this study, and replicating the evaluation design used in this study in other situations to advance the spread and scale of other tools and approaches for integrated care.

## Conclusion

The ODJJ shows promise for broad implementation and use across diverse settings to promote more integrated dementia care. The evaluation and resulting marketing strategy for scale and spread represent advancement in dementia care tool dissemination research by demonstrating how pilot implementations with deeply integrated users of various types can generate actionable plans for scale and spread of tools and resources.
